# BDNF rescues BAF53b-dependent synaptic plasticity and cocaine-associated memory in the nucleus accumbens

**DOI:** 10.1038/ncomms11725

**Published:** 2016-05-26

**Authors:** André O. White, Enikö A. Kramár, Alberto J. López, Janine L. Kwapis, John Doan, David Saldana, M. Felicia Davatolhagh, Yasaman Alaghband, Mathew Blurton-Jones, Dina P. Matheos, Marcelo A. Wood

**Affiliations:** 1 301 Qureshey Research Lab, Department of Neurobiology and Behavior, University of California, Irvine, California 92697, USA; 2 Center for the Neurobiology of Learning and Memory, Irvine, California 92697, USA; 3 Irvine Center for Addiction Neuroscience (ICAN), University of California, Irvine, California 92697, USA; 4 Institute for Memory Impairments and Neurological Disorders, University of California, Irvine, California 92697, USA; 5 Perelman School of Medicine at the University of Pennsylvania, Philadelphia, Pennsylvania 19104, USA; 6 Sue and Bill Gross Stem Cell Research Center, University of California, Irvine, California 92697, USA

## Abstract

Recent evidence implicates epigenetic mechanisms in drug-associated memory processes. However, a possible role for one major epigenetic mechanism, nucleosome remodelling, in drug-associated memories remains largely unexplored. Here we examine mice with genetic manipulations targeting a neuron-specific nucleosome remodelling complex subunit, BAF53b. These mice display deficits in cocaine-associated memory that are more severe in BAF53b transgenic mice compared with BAF53b heterozygous mice. Similar to the memory deficits, theta-induced long-term potentiation (theta-LTP) in the nucleus accumbens (NAc) is significantly impaired in slices taken from BAF53b transgenic mice but not heterozygous mice. Further experiments indicate that theta-LTP in the NAc is dependent on TrkB receptor activation, and that BDNF rescues theta-LTP and cocaine-associated memory deficits in BAF53b transgenic mice. Together, these results suggest a role for BAF53b in NAc neuronal function required for cocaine-associated memories, and also that BDNF/TrkB activation in the NAc may overcome memory and plasticity deficits linked to BAF53b mutations.

## Abstract (web summary)

Epigenetic mechanisms play a key role in drug-associated memories and behaviors. Here authors show that mice deficient of BAF53b, a nucleosome remodeling complex subunit, display deficits in synaptic plasticity and cocaine-associated memory, both of which can be rescued by BDNF application.

## Graphical abstract

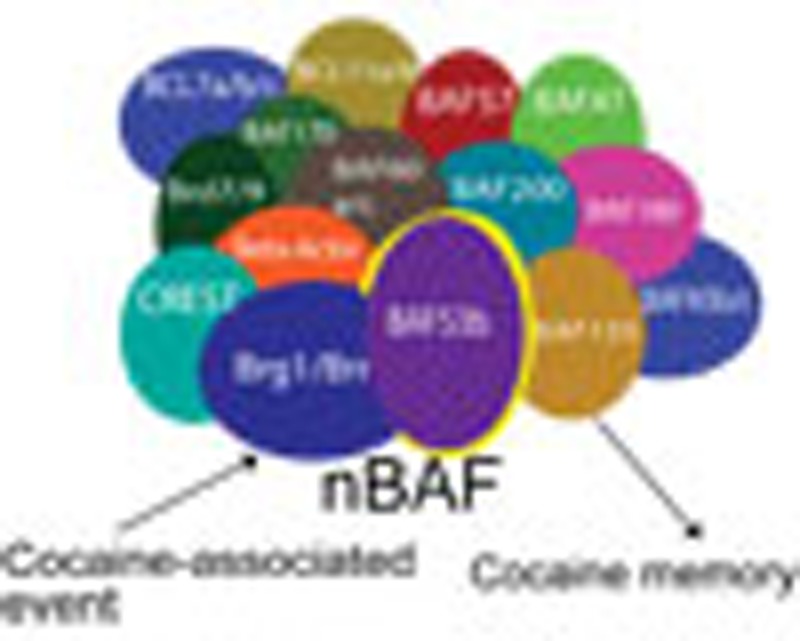

Drug addiction is characterized by compulsive drug-seeking behaviour despite negative consequences^1^. In the field of substance abuse, there remains unanswered questions about how drugs of abuse drive the establishment of long-lasting changes in the brain that support robust drug-associated behaviours and memories. A central feature of drugs of abuse that distinguishes them from non-addictive drugs is their ability to promote persistent changes in neuronal function in the reward pathways of the brain^2^,^3^,^4^. For example, cocaine is known to generate changes in histone modification patterns that result in aberrant gene expression profiles and synaptic plasticity in the nucleus accumbens (NAc) that drive several cocaine-seeking behaviours^5^,^6^,^7^,^8^,^9^,^10^,^11^.

Increasing evidence supports a role for epigenetic mechanisms in memory formation (for a review, see ref. 12). In particular, epigenetic mechanisms such as histone modification are integral for the formation of drug-associated memories and behaviours^13^,^14^. The basic repeating subunit of chromatin is the nucleosome, which comprises a DNA–histone complex that packages genomic DNA. Several epigenetic mechanisms including posttranslational histone modification, DNA methylation and non-coding RNA serve as key regulators of chromatin structure and gene expression that are required for aspects of drug-associated behaviours (see reviews^14^,^15^,^16^). For example, histone acetylation and methylation mechanisms are pivotal for cocaine-induced changes in gene expression that underlie cocaine-induced behaviours and cocaine-context-associated memories^6^,^7^,^8^,^9^,^10^,^11^,^17^,^18^. However, there is a fourth major epigenetic mechanism (ATP-dependent nucleosome remodelling) that regulates transcription by altering chromatin structure^19^. Currently, it remains unclear to what extent ATP-dependent nucleosome remodelling complexes are involved in drug-induced behaviours and drug-associated memories.

Nucleosome remodelling complexes are evolutionarily conserved multi-subunit complexes that replace, eject and alter nucleosomes to promote changes in gene expression^20^. Through their ATPase subunit, nucleosome remodelling complexes manipulate nucleosome–DNA contacts through ATP hydrolysis, thereby allowing the binding of the transcription machinery and associated transcriptional regulators^19^,^20^. A recent study identified the neuron-specific nucleosome remodeller, nBAF, as a regulator of gene expression necessary for neuronal differentiation^21^, dendritic branching^22^, hippocampal synaptic plasticity and hippocampus-dependent long-term memory^23^. nBAF contains one of two ATPase subunits (Brm or Brg1), which is necessary for ATP hydrolysis during nucleosome remodelling^24^. The nBAF complex also contains a unique subunit called *Baf53b* (also referred to as *Actl6b*), which appears critical for nBAF to target specific promoter regions^22^. BAF53b is only expressed in postmitotic neurons and only found in the nBAF complex^22^,^25^. The unusual dedication of BAF53b to the nBAF complex, as well its neuronal specificity, makes BAF53b the ideal target with which to examine the role of nucleosome remodelling complexes in cocaine-associated behaviours and memories. We examined whether BAF53b has a role in the formation of cocaine-associated behaviours and memories using mice that had either a deletion mutation or traditional heterozygous knockout of BAF53b.

The role of neuron-specific nucleosome remodelling factors in drug-associated memory formation and their influence on synaptic plasticity in the NAc has not been previously explored. Therefore, we extended our studies to examine the role of BAF53b in long-term potentiation (LTP) in NAc, as LTP is thought to be a candidate mechanism underlying long-term memory formation. First, we established a stimulation protocol that induced LTP using theta stimulation (theta-LTP) in slices from wild-type (WT) mice and compared the level of stable potentiation with that achieved in slices from BAF53b mutant mice. Next, we tested the possibility that (1) theta-LTP in the NAc requires TrkB receptor activation and (2) whether the ligand for TrkB, brain-derived neurotrophic factor (BDNF), could reverse deficits in theta-LTP and cocaine-associated memory observed in BAF53b mutant mice. Together, these experiments provide the first evidence for a role of neuron-specific nucleosome remodelling complexes in cocaine-associated memory formation and a potential mechanism for correcting related plasticity deficits in the NAc.

## Results

### Characterization of BAF53b mutant mice

**Figure 1:**
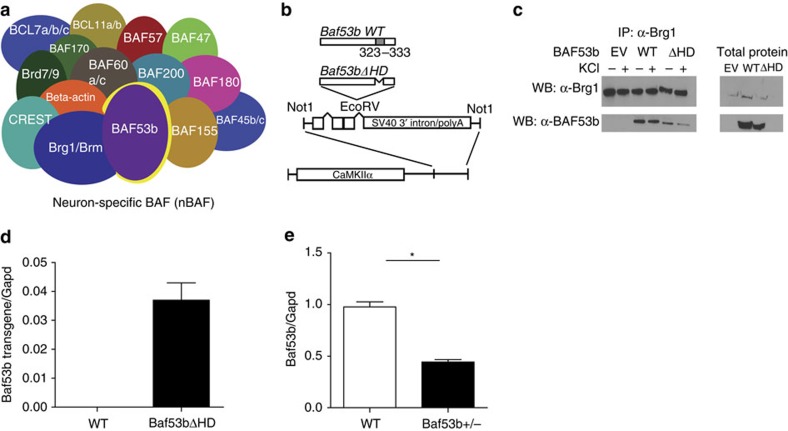
Characterization of *Baf53b*^*+/−*^ heterozygous knockout and *Baf53bΔHD* mice. (**a**) nBAF complex is combinatorially assembled, meaning that the individual subunits included in each unique complex can alter CRC function^20^ containing the dedicated and neuron-specific subunit BAF53b (highlighted in yellow). (**b**) WT *Baf53b* is illustrated with the hydrophobic domain (grey). Amino acids 323–333 in the hydrophobic domain were deleted to generate the BAF53bΔHD construct. This *Baf53bΔHD* mutant sequence was then cloned into a separate vector containing intron and exon sequences with splice sites and the SV40 intron and polyadenylation signal, which was then cloned downstream of the 8.5-kb mouse CamkIIα promoter^23^. This construct was then used to generate the *Baf53bΔHD* transgenic mice. (**c**) Immunoprecipitation and western blotting showing that Brg1 co-immunoprecipitates with BAF53b and BAF53bΔHD. (**d**) RT–qPCR revealed that the BAF53bΔHD transgene is only expressed in the BAF53bΔHD mutant mice and not in their WT littermates. Data are presented as mean±s.e.m. (**e**) RT–qPCR revealed that *Baf53b*^*+/−*^ heterozygous knockout mice (*n*=8) have significantly reduced WT *Baf53b* expression in the NAc compared with their WT littermates (*n*=7; *t*(13)=10.25, *P*<0.0001).

nBAF is a multi-subunit complex that is neuron specific (Fig. 1a). It is defined, in part, by the BAF53b subunit (highlighted in yellow), which is only found in the nBAF complex^22^,^25^. BAF53b is expressed in the NAc, a brain area required for cocaine-associated memory formation. BAF53b homozygous knockout mice (*Baf53b*^*−/−*^) do not survive into adulthood^22^. Therefore, to study the role of BAF53b in cocaine-associated memories and behaviours, we examined a deletion mutation of *Baf53b* (*Baf53bΔHD*). We previously demonstrated that a similar deletion of the hydrophobic domain of BAF53a (the non-neuronal homologue of BAF53b) generates a dominant negative form of BAF53a^26^. The transgene in the *Baf53bΔHD* line is expressed via the CaMKIIα promoter (Fig. 1b)^23^,^27^,^28^. Both WT BAF53b and the BAF53bΔHD mutant protein interact with Brg1, as demonstrated by co-immunoprecipitation of either WT or BAF53bΔHD with Brg1 (Fig. 1c). Using quantitative reverse-transcription PCR, we verified transgene expression in the NAc (Fig. 1d). In addition to transgenic *Baf53bΔHD* mice, we also examined a *Baf53b*^*+/−*^ heterozygous knockout mouse line^22^,^23^. *Baf53b*^*+/−*^ heterozygous mice have ∼50% *Baf53b* messenger RNA and protein expression in the hippocampus^23^. Quantitative reverse-transcription PCR revealed that *Baf53b* mRNA expression in the NAc of *Baf53b*^*+/−*^ heterozygous mice was significantly reduced (∼50%) compared with their WT littermates (Fig. 1e). Both genetically modified mouse lines have previously been shown to be indistinguishable from WT littermates with respect to locomotor activity, anxiety, short-term memory and basal synaptic transmission^21^.

### BAF53b mutant mice have normal cocaine sensitization

**Figure 2:**
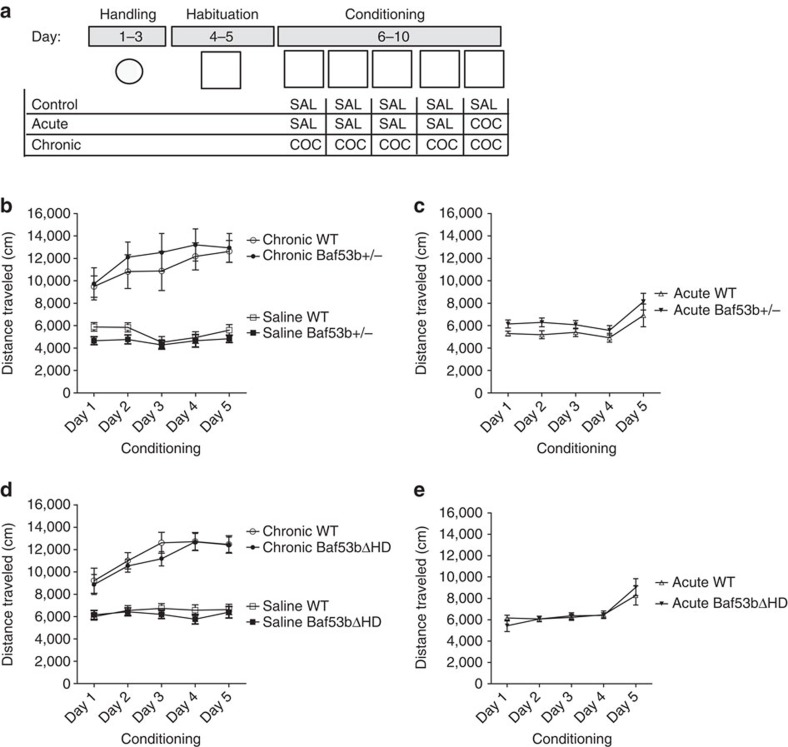
BAF53b mutant mice have normal cocaine sensitization. (**a**) Schematic representation of cocaine sensitization procedure. (**b**) Throughout conditioning, chronic cocaine-treated *Baf53b*^*+/−*^ heterozygous knockout mice (*n*=8) travelled the same mean distance ±s.e.m. as chronic cocaine-treated WT littermates (*n*=8; significant main effect of injection day, F_4,56_=13.90, *P*<0.0001; no main effect of genotype, F_1,14_=0.24, *P*=0.6; no interaction, F_4,56_=0.78, *P=*0.54). (**c**) Acute cocaine-treated *Baf53b*^*+/−*^ heterozygous knockout mice (*n*=9) travelled the same distance as acute cocaine-treated WT littermates (*n*=8; significant main effect of injection day, F_4,60_=11.78, *P<*0.0001; no main effect of genotype, F_1,15_=2.70, *P*=0.12; no interaction, F_4,60_=0.24, *P*=0.92). (**d**) Both *Baf53bΔHD* and WT mice had a similar response to chronic cocaine administration with both groups showing a graded increase in their response to cocaine indicative of cocaine sensitization (significant main effect of injection day, F_4,52_=20.54, *P<*0.0001; no main effect of genotype, F_1,13_=0.23, *P=*0.64; no interaction, F_4,52_=0.76, *P=*0.56). (**e**) Similarly, no differences were observed between *Baf53bΔHD* and WT mice after acute cocaine administration (significant main effect of injection day, F_4,52_=14.46, *P*<0.0001; no main effect of genotype, F_1,13_=0.01, *P=*0.94; no interaction, F_4,52_=0.78, *P=*0.55).

We investigated the role of BAF53b in cocaine sensitivity. We and other laboratories have shown that histone-modifying enzymes can play a role in cocaine-induced sensitization and responses to repeated cocaine administration^6^,^7^,^18^,^29^. Therefore, to test whether BAF53b plays a role in cocaine sensitivity, *Baf53b*^*+/−*^ heterozygous mice were given acute or chronic cocaine treatments followed by assessment of locomotor activity (Fig. 2a). A 10-mg kg^−1^ cocaine dose, which generates robust sensitization, was used as in our previous experiments (ref. 6). *Baf53b*^*+/−*^ heterozygous mice were assessed for locomotor deficits (Supplementary Fig. 1a). Heterozygous knockout of BAF53b did not affect baseline locomotor activity during habituation (Supplementary Fig. 1b). In addition, both *Baf53b*^*+/−*^ heterozygous and WT mice had similar responses to chronic cocaine administration, with both groups showing a graded increase in their response to cocaine indicative of cocaine sensitization (Fig. 2b). Similarly, no differences were observed between *Baf53b*^*+/−*^ heterozygous and WT mice after acute cocaine administration (Fig. 2c). Therefore, we found that the loss of one allele of *Baf53b* did not alter cocaine sensitivity, as *Baf53b*^*+/−*^ heterozygous mice performed comparably with WT littermates.

Similarly, we investigated whether the deletion mutant of BAF53b would affect cocaine sensitivity. *Baf53bΔHD* mice were given acute or chronic cocaine treatments and their locomotor activity recorded in the same manner as the *Baf53b*^*+/−*^ heterozygous mice. A 10-mg kg^−1^ cocaine dose was used as in the previous experiment. *Baf53bΔHD* mice displayed normal baseline locomotor activity during habituation (Supplementary Fig. 1c). Both *Baf53bΔHD* and WT mice had a similar response to chronic cocaine administration indicative of cocaine sensitization (Fig. 2d). Similarly, no differences were observed between *Baf53bΔHD* and WT mice after acute cocaine administration (Fig. 2e). Therefore, we found that the deletion mutation did not alter cocaine sensitivity, as *Baf53bΔHD* mice performed comparably with WT littermates. Overall, these results suggest that BAF53b is not required for cocaine sensitization.

### BAF53b is required for cocaine-associated memory

**Figure 3:**
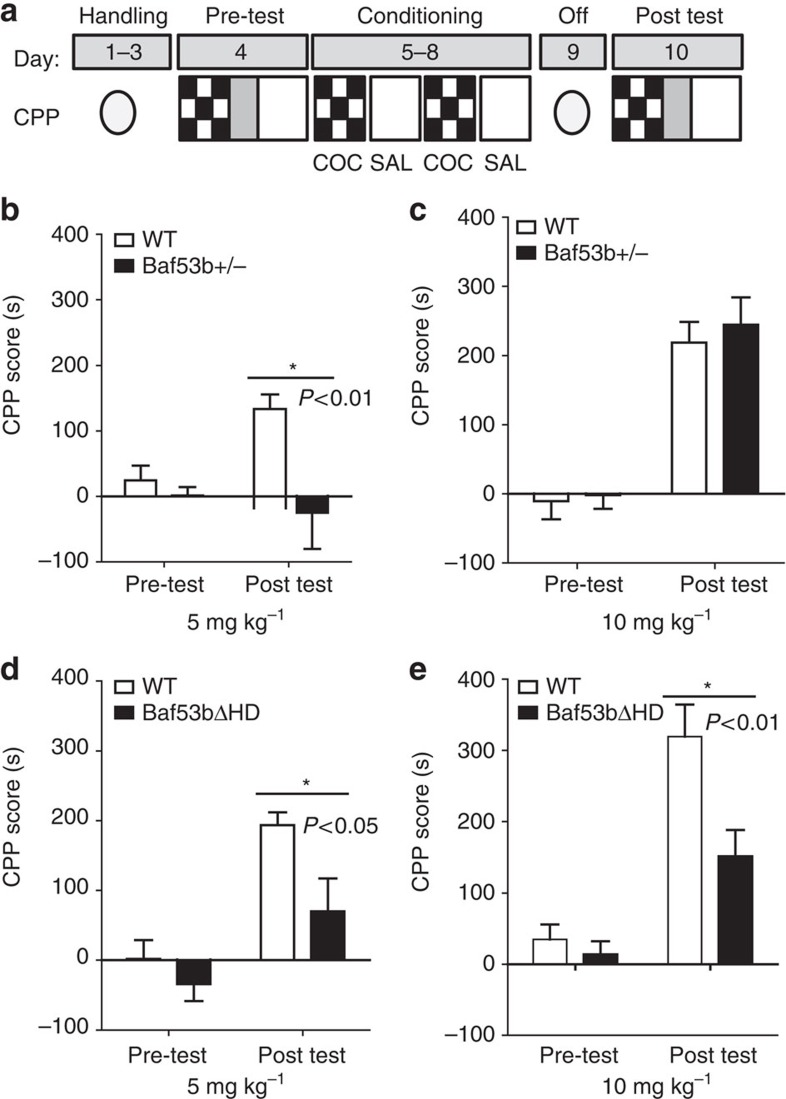
Mutant mice have impairments in cocaine CPP. (**a**) Schematic representation of cocaine-CPP procedure. (**b**) Cocaine-CPP expression indicated by mean CPP score (CS+ minus CS−)±s.e.m. At 5 mg kg^−1^ cocaine dose, *Baf53b*^*+/−*^ heterozygous knockout mice (*n*=10) exhibited significantly attenuated CPP score compared with WT littermates (*n*=8). A two-way repeated-measures analysis of variance (ANOVA) revealed a significant main effect of genotype (F_1,16_=6.99, *P*=0.02). No effect of conditioning was observed (F_1,16_=2.05, *P*=0.17) but there was a significant interaction (F_1,16_=4.93, *P*=0.04). Bonferroni *post-hoc* analysis showed no initial preference for either context on the pretest (*t*=0.46, *P>*0.05). However, there was a significant difference between the WT and *Baf53b*^*+/−*^ heterozygous mice on the posttest (*t*=3.45, *P*<0.01). (**c**) At 10 mg kg^−1^ cocaine dose, *Baf53b*^*+/−*^ heterozygous knockout mice (*n*=9) exhibit similar CPP score to WT littermates (*n*=8). Using a two-way repeated-measures ANOVA, we found a significant main effect of conditioning (F_1,17_=112.81, *P<*0.0001) but not genotype (F_1,17_=0.24, *P=*0.63) and no interaction (F_1,17_=0.14, *P*=0.71). Bonferroni *post-hoc* analysis showed no difference between the WT and the *Baf53b*^*+/−*^ heterozygous mice on the pretest (*t*=0.21, *P>0.05*) or posttest (*t*=0.61, *P>*0.05). (**d**) At 5 mg kg^−1^ cocaine dose, *Baf53bΔHD* mice (*n*=9) exhibited significantly attenuated CPP score compared with WT littermates (*n*=10). Using a two-way repeated-measures ANOVA, we found significant main effects on conditioning (F_1,17_=29.19, *P<*0.0001) and genotype (F_1,17_=5.62, *P=*0.03), but no interaction (F_1,17_=2.56, *P=*0.13). Bonferroni *post-hoc* analysis showed no differences between the preference of WT and *Baf53bΔHD* mice for either context on the pretest (*t*=0.83, *P>*0.05) but a significant difference on the posttest (*t*=2.85, *P<*0.05). (**e**) At a 10-mg kg^−1^ cocaine dose, *Baf53bΔHD* mice (*n*=10) exhibited significantly attenuated CPP score compared with WT littermates (*n*=7). A two-way repeated-measures ANOVA revealed significant main effects of conditioning (F_1,15_=47.37, *P<*0.0001), genotype (F_1,15_=7.91, *P=*0.01) and an interaction (F_1,15_=5.71, *P*=0.03). Bonferroni *post-hoc* analysis showed no differences between the preference of WT and BAF53bΔHD.

To examine the role of BAF53b in cocaine-associated memory formation, we used cocaine-induced conditioned place preference (CPP). The CPP paradigm involves animals associating the rewarding properties of cocaine with a distinct cocaine-paired context compared with a distinct vehicle-paired context (Fig. 3a). The strength of the association is then tested while the animal is in a drug-free state. In the first experiment, *Baf53b*^*+/−*^ heterozygous mice and WT littermates were conditioned using 5 mg kg^−1^ cocaine (Fig. 3b). Neither *Baf53b*^*+/−*^ heterozygous mice nor WTs displayed an initial preference for either context before conditioning (pre-test). After conditioning with two pairings of cocaine, post-test results revealed that WT mice had a robust CPP score (time spent in cocaine-paired chamber minus saline-paired chamber). In contrast, *Baf53b*^*+/−*^ heterozygous mice did not display a cocaine-associated CPP, spending similar time in both cocaine-paired and saline-paired chambers after conditioning. We examined the locomotor activity during the test days to assess whether there were any differences in the locomotor activating effect of cocaine in *Baf53b*^*+/−*^ heterozygous mice compared with WT mice (Supplementary Fig. 2a). We observed no differences in locomotor activity between *Baf53b*^*+/−*^ heterozygous mice and WT controls on either test day (Supplementary Fig. 2b). These results suggest that the loss of one allele of *Baf53b* significantly disrupts cocaine CPP at 5 mg kg^−1^ without altering overall locomotor performance.

Next, we were interested in determining whether there was a dose-dependent role for BAF53b in cocaine CPP. Therefore, we conducted a separate CPP experiment at 10 mg kg^−1^ cocaine using a different cohort of *Baf53b*^*+/−*^ heterozygous mice (Fig. 3c). Similar to the previous CPP experiment, neither *Baf53b*^*+/−*^ heterozygous mice nor WT mice displayed an initial preference for either context. At post test, both WT and *Baf53b*^*+/−*^ heterozygous mice established a preference for the cocaine-paired context. Therefore, unlike the 5-mg kg^−1^ CPP experiment, one *Baf53b* allele is sufficient for cocaine CPP when using a 10-mg kg^−1^ cocaine dose. We also examined the locomotor activity during the 10-mg kg^−1^ experiment, to assess whether there were any differences in the locomotor-activating effect of cocaine in the *Baf53b*^*+/−*^ heterozygous mice compared with WT mice at 10 mg kg^−1^ (Supplementary Fig. 2c). However, no differences were observed between *Baf53b*^*+/−*^ heterozygous and WT mice on test days. Together, these results suggest that removal of one *Baf53b* allele disrupts cocaine CPP memory formation in a cocaine dose-dependent manner (at 5 mg kg^−1^ but not at 10 mg kg^−1^). Importantly, all *Baf53b*^*+/−*^ heterozygous mice travelled similar distances to their respective WT controls on test days, indicating no differential effects of cocaine on locomotor performance.

We then examined whether *Baf53bΔHD* mice had similar memory deficits to *Baf53b*^*+/−*^ heterozygous mice using a CPP experiment (Fig. 3d). Neither *Baf53bΔHD* mice nor WT mice displayed an initial preference for either context on the pretest. *Baf53bΔHD* mice and WT littermates were conditioned using 5 mg kg^−1^ cocaine. After conditioning, WT mice acquired a robust CPP, whereas *Baf53bΔHD* mice displayed a significantly reduced CPP. We observed no differences in locomotor activity between *Baf53bΔHD* mice and WT controls on either test day (Supplementary Fig. 2d). These findings further suggest that BAF53b is important for the formation of cocaine CPP at 5 mg kg^−1^.

To evaluate whether there was a dose-dependent role of BAF53b in cocaine CPP, we performed a 10-mg kg^−1^ cocaine CPP experiment in a different cohort of *Baf53bΔHD* mice (Fig. 3e). Similar to the previous experiment, neither *Baf53bΔHD* mice nor WT mice displayed an initial preference for either context before conditioning. After conditioning, WT mice displayed a robust preference for the cocaine-paired compartment. However, unlike the *Baf53b*^*+/−*^ heterozygous mice at 10 mg kg^−1^, *Baf53bΔHD* mice demonstrated a significantly reduced CPP post test. These results indicate that the *Baf53bΔHD* mutation disrupts the formation of cocaine-associated CPP in both doses of cocaine (at 5 and 10 mg kg^−1^). No differences in locomotor activity were observed between *Baf53bΔHD* mice and their WT littermates during the 10-mg kg^−1^ experiment (Supplementary Fig. 2e). Therefore, CPP deficits observed in the *Baf53bΔHD* mice appear to be independent of any locomotor impairment. Together, these data from the *Baf53bΔHD* and *Baf53b*^*+/−*^ heterozygous mice suggest that BAF53b plays a key role in the acquisition/consolidation of cocaine-associated CPP. However, there remains the open question of what accounts for the more severe cocaine-memory impairments observed in the *Baf53bΔHD* mice compared with the *Baf53b*^*+/−*^ heterozygous mice.

### BAF53bΔHD mice have deficits in synaptic plasticity

**Figure 4:**
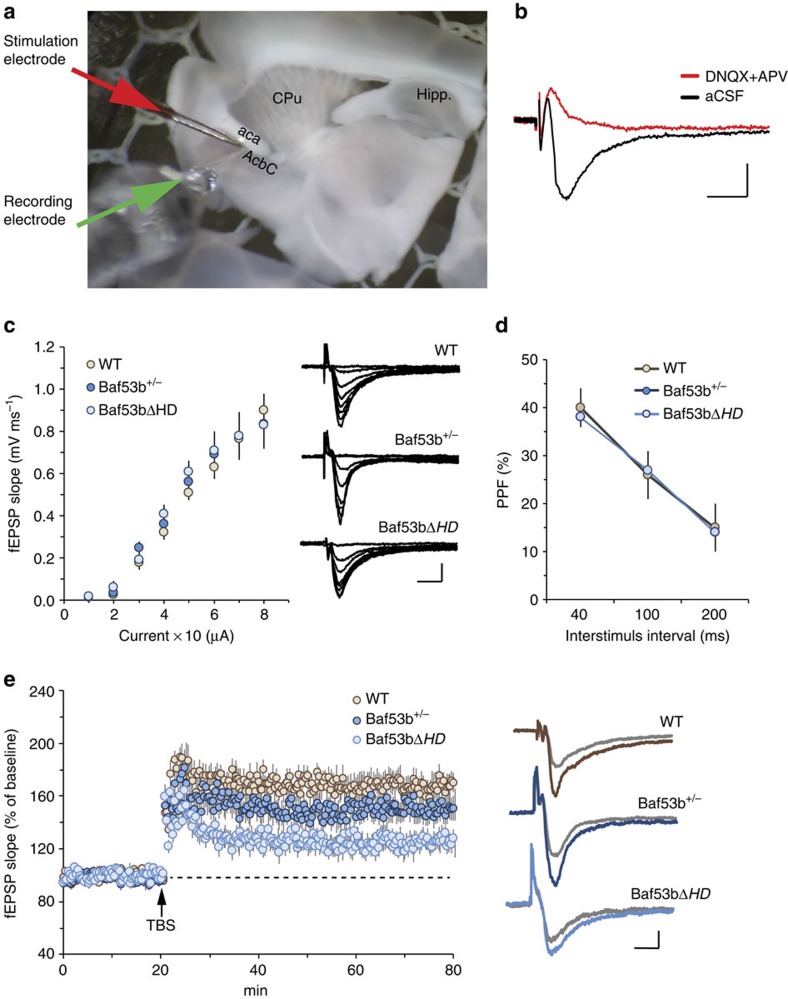
Mutation of BAF53b disrupts LTP in NAc core slices. (**a**) Photomicrograph of a parasagittal slice containing NAc and the relative position of the stimulation (red arrow) and recording (green arrow) electrodes (1.08 lateral to mid-sagittal plane).aca, anterior commissure; AcbC, accumbens nucleus core; CPu, caudate putamen; Hipp., hippocampus. (**b**) Representative trace of glutamatergic transmission collected within the NAc (black line) that is blocked by AMPA and NMDA receptor antagonists, 20 μM DNQX and 50 μM D-APV, respectively (red line). Scale bar, 0.2 mV, 10 ms. (**c**) Left, input/output curves measuring the slope of the fEPSP response across a range of stimulation currents (10–80 μA) was comparable between *Baf53b*^*+/−*^ (dark blue circles, *n*=9), *Baf53bΔHD* (light blue circles, *n*=9) and WT (WT) (brown circles, *n*=9) mice (F_2,24_=0.15, *P=*0.81). Right, representative traces collected during the generation of input/output curves from WT, *Baf53b*^*+/−*^ and *Baf53bΔHD* slices (scale bar, 0.2 mV per, 5 ms). (**d**) Paired-pulse facilitation of the initial slope of the synaptic response was comparable (40, 100 and 200 ms inter-pulse intervals) in slices from *Baf53b*^*+/−*^ (blue circles, *n*=9), *Baf53bΔHD* (light blue circles, *n*=10) and WT (brown circles, *n*=8) mice (F_2,24_=0.16, *P=*0.96). (**e**) Left, LTP induced using TBS (black arrows) produced stable potentiation in slices from WT mice (brown circles). Short-term potentiation measured 5 min post TBS was no different between groups (F_2,25_=6.22, *P*=0.11). Although potentiation measured 50–60 min post TBS was marginally reduced in slices from *Baf53b*^*+/−*^ (blue circles, *n*=9), LTP in slices from *Baf53bΔHD* mice (light blue circles, *n*=10) was significantly impaired relative to control WT slices (brown circles, *n*=9) (F_2,25_=31.79, *P=*0.0013). Right, fEPSP traces collected during baseline (grey line) and 1 h after TBS (coloured line). Scale bar, 0.2 mV per, 5 ms.

The observed differences in cocaine-associated memory between the *Baf53bΔHD* and *Baf53b*^*+/−*^ heterozygous mouse lines led us to investigate whether synaptic plasticity was differentially altered. One of the likeliest candidates for how associative memories are generated is through changes in synaptic plasticity. Brain regions within the reward pathway such as the NAc display LTP and long-term depression^30^,^31^; however, it remains unknown as to how disruption of BAF53b within the NAc influences long-term synaptic plasticity in that region. Thus, we conducted a study using parasagittal slices containing the NAc core from (1) *Baf53b*^*+/−*^ heterozygous, (2) *Baf53bΔHD* and (3) WT littermate mice, to examine BAF53b influences on LTP (Fig. 4a).

First, we established that the field excitatory postsynaptic potentials generated within the core following afferent stimulation of glutamatergic fibres that form synapses on medium spiny neurons were blocked by AMPA (α-amino-3-hydroxy-5-methyl-4-isoxazole propionic acid) and NMDA (*N*-methyl-D-aspartate) receptor antagonists, DNQX and D-APV, respectively (Fig. 4b). Low-intensity, single pulse stimulation of afferent fibres within the core produced similar field responses to those described in previous studies^32^,^33^,^34^. Next, we generated input/output curves to determine whether the slope of the field excitatory postsynaptic potential (fEPSP) responses across a range of stimulation currents differed between the two genetically modified mice relative to WT controls (Fig. 4c). No differences were found between groups. Next, we tested for differences in transmitter release kinetics using paired-pulse facilitation. The *Baf53b*^*+/−*^ heterozygous and *Baf53bΔHD* slices did not differ from WT slices (Fig. 4d). Taken together, these results indicated that genetic manipulation of BAF53b did not alter conventional measures of baseline synaptic transmission in the NAc core.

We then examined LTP using theta burst stimulation (TBS). Trains of five theta bursts, which has been previously shown to be the threshold for inducing stable potentiation in mice^23^, produced a robust and immediate potentiation in WT slices, which briefly decayed over a 10–15-min period, to stabilize at ∼70% above the pre-TBS baseline (Fig. 4e). Slices containing NAc core from *Baf53b*^*+/−*^ heterozygous mice exhibited normal short-term potentiation that was no different from WT values but the level of LTP 60 min post TBS was slightly attenuated relative to controls. In contrast, TBS delivered to *Baf53bΔHD* slices produced a profound deficit in LTP. Short-term potentiation was comparable to WT controls, but the level of potentiation 60 min post TBS was remarkably reduced compared with WT controls. Collectively, these findings suggest that BAF53b plays a pivotal role in the cellular processes that maximize synaptic strength in the NAc. This result is particularly pronounced in the *Baf53bΔHD* slices and correlates with the greater cocaine-associated memory impairment observed in this transgenic mutant line (Fig. 3).

### BDNF reverses LTP and CPP deficits in BAF53bΔHD mice

In an effort to restore deficits in cocaine memory and synaptic plasticity, we explored the role of BDNF in the NAc. BDNF is a potent positive neuromodulator in the brain and has been reported to play an important role in influencing addictive-type behaviours (see reviews^35^,^36^). Given that BDNF is released from terminals following TBS^37^,^38^ and effectively facilitates LTP^39^,^40^,^41^ and memory in rodents^42^, we tested the possibility that increasing exogenous levels of BDNF in the slice would reverse the impairment in LTP in *Baf53bΔHD* slices.

**Figure 5:**
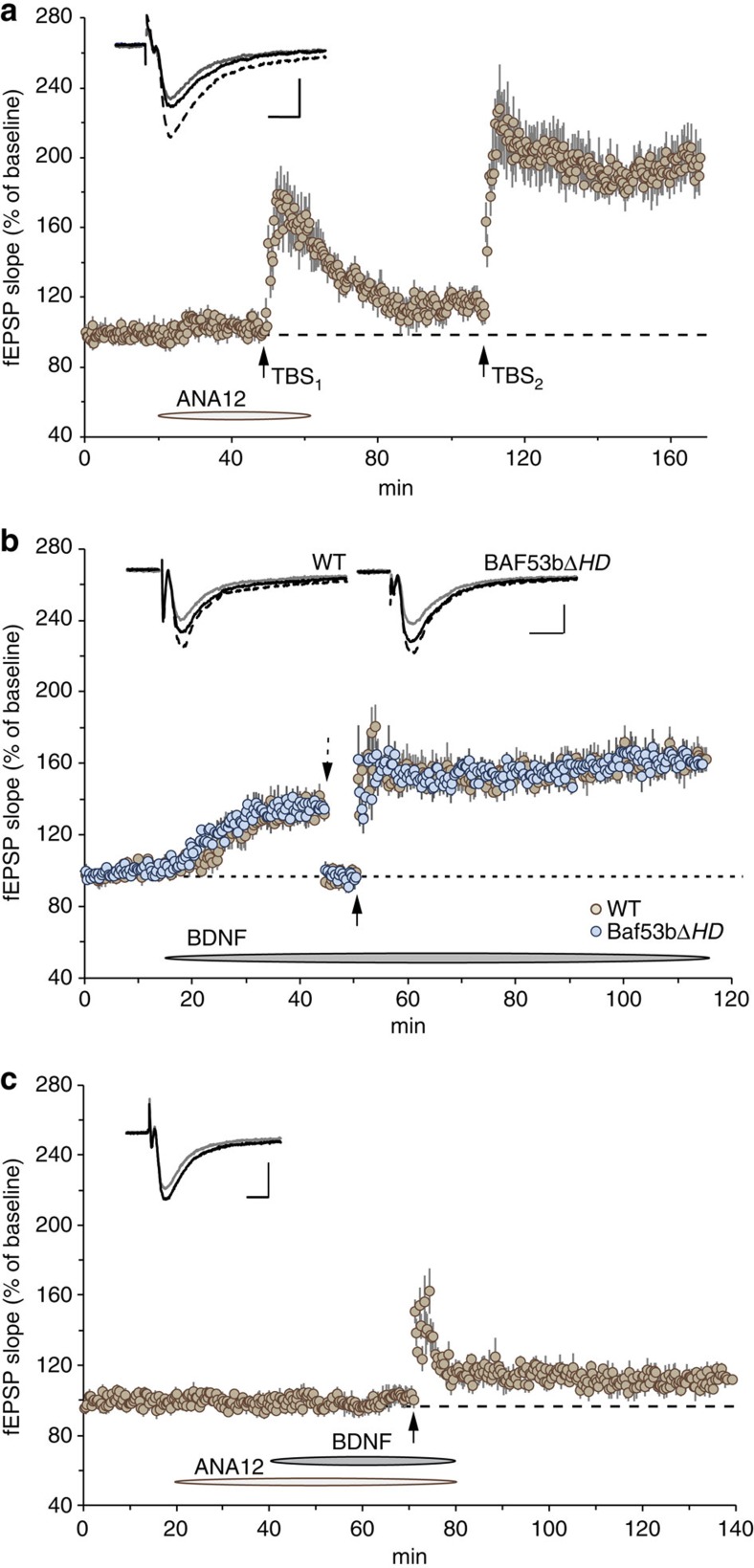
Infusions of BDNF reverse LTP deficit in *Baf53bΔHD* slices. (**a**) Bath application of 500 nM ANA12 blocked theta burst-induced LTP (TBS_1_) in NAc (*n*=6) in slices from WT mice. A 60-min period of ANA12 washout restored LTP after a second bout of theta stimulation (TBS_2_) in the same stimulated pathway. Inset, representative traces collected during baseline (grey line), 60 min post-TBS_1_ (blue line) and 60 min post-TBS_2_ (dark blue line). Scale bar, 0.4 mV, 5 ms. (**b**) Infusion of BDNF (2 nM) began after a 15-min stable baseline recording. BDNF produced a rapid increase in glutamatergic transmission (∼35%) within the NAc in slices from both WT (*n*=6) and *Baf53bΔHD* (*n*=5) mice. Before delivery of theta bursts, stimulation intensity was reduced to reset baseline to pre-drug levels, to evaluate the effects of BDNF on the magnitude of LTP (dotted downward arrow). Five theta trains (five theta bursts each, separated by 1 min) delivered 5 min after re-establishing stable baseline recordings (upward arrow) produced robust and stable LTP in WT slices and completely reversed the LTP impairment previously seen in slices from *Baf53bΔHD* mice (see Fig. 4e). Inset, representative traces collected during baseline (grey line), 30 min after start of BDNF infusion (blue line) and 60 min post TBS (dark blue line). Scale bar. 0.4 mV, 5 ms. (**c**) Pretreatment with 500 nM ANA12 completely blocked BDNF-induced increases in baseline synaptic transmission. The delivery of TBS produced a brief short-term potentiation that rapidly returned towards baseline levels (*n*=6). Inset, representative traces collected during baseline (grey line) and 60 min post TBS (black line). Scale bar, 0.5 mV, 5 ms.

It was critical to first establish whether LTP in the NAc was BDNF/TrkB receptor dependent. We used a novel TrkB receptor antagonist, ANA12 (ref. 43), to test whether blocking TrkB receptor activation would inhibit theta-LTP. As summarized in Fig. 5a, the compound had no measureable influence on baseline synaptic transmission elicited by stimulation of afferent glutamatergic fibres, but effectively blocked theta burst-induced LTP. Following a 60-min washout of the drug, a second set of theta stimulation produced stable LTP, suggesting that the compound is fully reversible. These data indicate that TrkB activation has a role in the consolidation of LTP in the NAc.

We then examined whether increasing exogenous levels of BDNF in the slice preparation would alter baseline synaptic transmission and reverse LTP impairment in *Baf53bΔHD* slices. Administration of recombinant BDNF generated a rapid increase in fEPSP slope in NAc slices from WT and *Baf53bΔHD* mice (Fig. 5b). This effect was significantly above baseline within 30 min after the start of BDNF infusion. Stimulation intensity was then adjusted to pre-drug baseline levels and TBS was applied to determine the magnitude of LTP in the presence of BDNF for both *Baf53bΔHD* and WT controls. Both groups displayed robust potentiation that remained stable for the remainder of the recording period. In a follow-up experiment, we were interested in determining whether the BDNF-induced increase in synaptic transmission and LTP were dependent on TrkB receptor activation. Slices from WT mice were pretreated with the ANA12 compound 20 min before a 30 min co-application of BDNF followed by TBS. As shown in Fig. 5c, the TrkB receptor antagonist blocked BDNF-induced increases in baseline synaptic transmission and theta-LTP. These data are similar to a previous report, indicating that BDNF release promotes LTP in the dorsal striatum^44^. Taken together, BDNF appears to function as a neuromodulator at synapses on medium spiny neurons within the NAc core and activating its receptor, TrkB, circumvents the BAF53bΔHD-induced LTP deficits by producing potentiation levels comparable to control levels as observed in WT slices.

**Figure 6:**
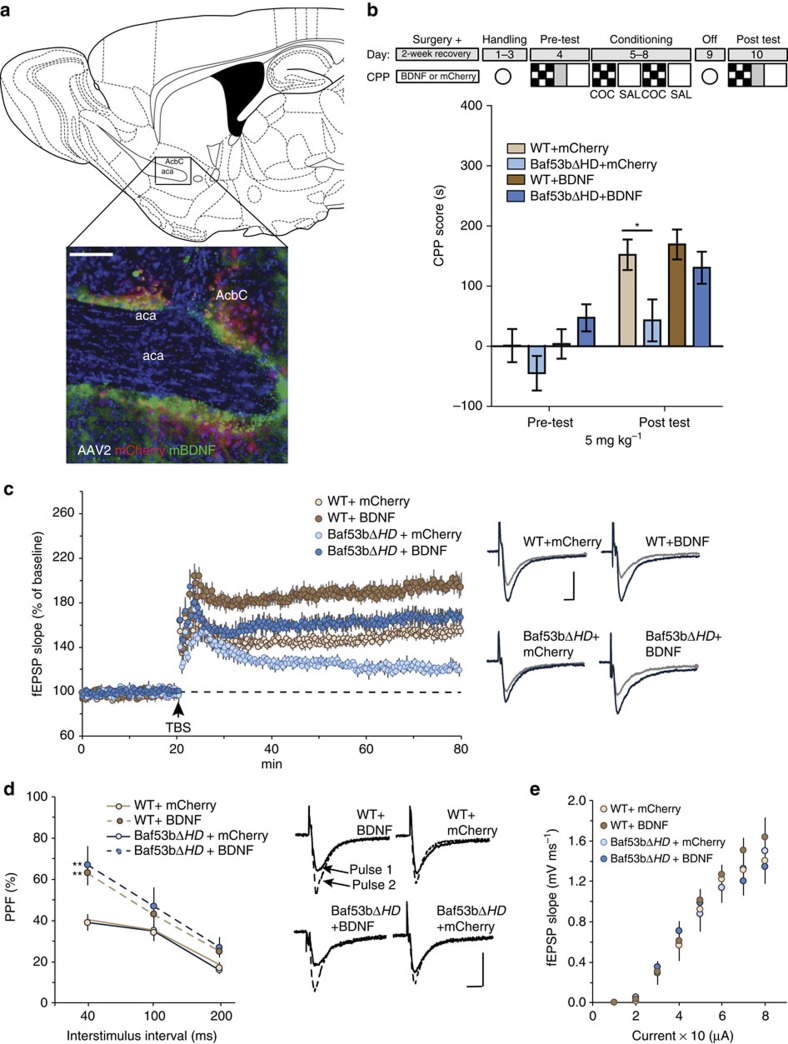
Viral overexpression of BDNF reverses CPP and LTP deficits in *Baf53bΔHD* mice. (**a**) Immunohistological image showing viral expression in the NAc using mCherry (red), BDNF (green) and DAPI (blue). Scale bar, 100 μm. Schematic representation of cocaine-CPP procedure. (**b**) Cocaine-CPP expression indicated by mean CPP score (CS+versus CS−)±s.e.m. At 5 mg kg^−1^ cocaine dose, WT+mCherry (*n*=15) and WT+BDNF (*n*=16) robust CPP. *Baf53bΔHD*+BDNF (*n*=12) established a CPP comparable to WT groups. *Baf53bΔHD*+mCherry (*n*=14) exhibited significantly attenuated CPP score compared with WT groups. A two-way repeated-measures analysis of variance (ANOVA) revealed a significant main effect of group (F_3,53_=3.08, *P*=0.035) and conditioning (F_3,53_=95.10, *P*<0.0001) There was a significant interaction (F_3, 53_=2.90, *P*=0.043). Bonferroni *post-hoc* analysis showed no initial preference for either context on the pretest (*t*=2.861, *P<*0.05). (**c**) Left, the initial expression of LTP was significantly greater in WT+BDNF slices (*n*=7) relative to WT+mCherry (*t*_15_=2.32, *P*=0.018) slices (*n*=10), as was the level of potentiation 60 min after TBS in the former group (*t*_15_=6.95, *P*<0.0001). In contrast, slices (*n*=9) from *Baf53bΔHD*+BDNF mice produced a similar level of short-term potentiation as found in slices (*n*=8) from *Baf53bΔHD*+mCherry mice (*t*_15_=1.14, *P*=0.13); however, virally expressing BDNF in *Baf53bΔHD* mice rescued the impairment in LTP found in slices from mice expressing the control mCherry-linked virus (*t*_15_=6.10, *P*<0.0001). There was no significant difference in the level of potentiation at 60 min post TBS between WT+mCherry and *Baf53bΔHD*+BDNF slices (*t*_17_=*P*=0.09). Right, representative traces collected during initial baseline recordings (grey line) and 60 min post TBS (black line) for each group. Scale bar, 5 ms per, 0.5 mV. (**d**) Left, virally expressing BDNF caused a significant increase in paired-pulse facilitation (PPF) at the shortest interval tested (40 ms) in slices from both WT and *Baf53bΔHD* mice (F_3,30_=13.8, *P*=0.025). Right, representative examples of PPF recorded at 40 ms interstimulus interval for each group tested. Traces of the first (solid black line) and second (dotted black line) fEPSPs are superimposed. Scale bar, 5 ms per, 0.5 mV. (**e**) Input/Output curves measuring the slope of the fEPSP response across a range of stimulation currents (10–80 μA) was comparable among all groups (F_3,30_=0.25, *P*=0.87).

Given that exogenous recombinant human BDNF was sufficient to rescue theta-LTP deficits in *Baf53bΔHD* slices, we extended these findings by testing whether viral delivery of human BDNF into the NAc would rescue the CPP deficits (Fig. 6a). We infused either AAV2-mCherry-BDNF or AAV2-mCherry (control) into the NAc of WT (WT+BDNF; WT+mCherry) and *Baf53bΔHD* (*Baf53bΔHD*+BDNF; *Baf53bΔHD*+mCherry) mice. After a 2-week recovery period, we evaluated each group's capacity for cocaine-associated memory formation using CPP (Fig. 6b). No group displayed a significant chamber preference on the pretest. At post test, both WT+BDNF and WT+mCherry groups displayed a robust CPP. The *Baf53bΔHD*+mCherry group failed to establish a CPP, as predicted. In contrast, the *Baf53bΔHD*+BDNF group displayed a preference for the cocaine-paired chamber comparable to the WT groups. These results suggest that viral delivery of human BDNF rescued the CPP impairments in this mutant line.

We later evaluated the effects of the BDNF virus on synaptic function in the NAc using a subset of mice from each group tested in CPP. We found that theta-induced potentiation in slices from control mice (WT+mCherry) was similar to that found in slices from WT mice without any virus (see Fig. 4e); however, slices from BDNF-expressing control mice (WT+BDNF) produced a dramatic increase in potentiation, minutes following LTP induction and 60 min after TBS (Fig. 6c). We then examined slices from *Baf53bΔHD*+mCherry group and, as predicted, LTP was significantly reduced 60 min post induction. In accordance with the behavioural results, LTP was rescued in slices taken from the *Baf53bΔHD*+BDNF group (Fig. 6c). We were also interested in determining whether chronic exposure to BDNF may have altered presynaptic release. As shown in Fig. 6d, both groups injected with the BDNF virus (WT+BDNF and *Baf53bΔHD*+BDNF) showed a significant increase in paired-pulse facilitation at the shortest interpulse interval tested as compared with their respective control vehicle groups (WT+mCherry and *Baf53bΔHD*+mCherry). Finally, we also measured for changes in neuronal excitability between groups by generating an input/output curve. We found no difference between groups across a range of stimulation intensities (Fig. 6e). The results of our electrophysiological and behavioural tests indicate that viral infusion of human BDNF is sufficient to rescue cocaine-associated memory and synaptic function in *Baf53bΔHD* mutant mice. Collectively, our results suggest that manipulation of BDNF/TrkB receptor activation can overcome the cocaine memory and plasticity deficits caused by deleting the hydrophobic domain of BAF53b.

## Discussion

Understanding how drugs of abuse alter neuronal function in the reward pathway can provide insight into how these drugs support the formation of persistent drug-associated memories. Emerging molecular and cellular research has established roles for epigenetic mechanisms in drug-induced behaviours and drug-associated memory formation. In the current study, we investigated the role of the neuron-specific nucleosome remodelling subunit BAF53b in cocaine-associated memory formation and synaptic plasticity. Genetic manipulations targeting the BAF53b subunit produced severe impairments in cocaine-associated memory and synaptic plasticity. Moreover, the degree of deficits observed in cocaine-associative memory correlated with the degree of impairments observed in theta-LTP in the NAc of mutant mice. These deficits in theta-LTP and CPP were rescued using BDNF administration. Together, these results demonstrate a novel role for BAF53b in cocaine-associated memory formation and synaptic plasticity in the NAc.

Although histone modification and nucleosome remodelling can work in a coordinated manner to regulate gene expression and the role of histone acetylation and methylation have been shown to have a critical role in cocaine action, until now there was no direct evidence linking a specific nucleosome remodeller in cocaine-associated behaviours. One previous study implicated the ATPase subunit Brg1 as being associated with cocaine-induced gene expression changes in rodents^5^. More specifically, Brg1 was shown to physically interact with the cyclin-dependent kinase 5 (*Cdk5*) gene during chronic cocaine conditions^5^. This was an enticing demonstration that Brg1 and nucleosome remodelling may be involved in cocaine-induced regulation of gene expression. However, that study did not link changes in Brg1 association to changes in cocaine-associated behaviour. Furthermore, targeting the role of Brg1 in cocaine-associated behaviours is confounded by the role of Brg1 as the ATPase in several different nucleosome remodelling complexes that are found in multiple cell types^19^.

By targeting BAF53b, we circumvented several of these limitations that prevent elucidating the role of nucleosome remodelling in cocaine-associative memory formation. BAF53b is unique, because it is currently thought to be exclusively found in the nBAF nucleosome remodelling complex and is neuron specific^22^,^25^. Examining a homozygous deletion of BAF53b (*Baf53b*^*−/−*^) was not possible, as those mice die shortly after birth^22^. Cultured neurons from homozygous knockout mice display aberrant gene expression, synapse formation and activity-dependent dendritic arborization^22^. Therefore, we used two independently derived mouse lines that avoid the lethality associated with BAF53b^*−*/*−*^ knockout mice. Both heterozygous knockout (*Baf53b*^*+/−*^) and *Baf53bΔHD* transgenic mice appear indistinguishable from WT littermates in development, anxiety, locomotor activity and short-term memory^23^. To further account for unforeseen effects associated with having only one *Baf53b* allele (heterozygous knockout) throughout development, the *Baf53bΔHD* transgenic line expresses mutant BAF53b outside of BAF53b's known role in embryonic development.

Even though both mutant mouse lines displayed profound deficits in cocaine-associated memory formation, their responses to an increase in cocaine dose differed. Increasing the dose of cocaine from 5 to 10 mg kg^−1^ resulted in a continued CPP impairment in the *Baf53bΔHD*, but not the *Baf53b*^*+/−*^ heterozygous mice. The lack of impairment in the *Baf53b*^*+/−*^ heterozygous knockout at 10 mg kg^−1^ was not predicted. In addition, both the *Baf53bΔHD* and *Baf53b*^*+/−*^ heterozygous mice travelled similar distances to their respective WT littermates at the 10-mg kg^−1^ dose. This suggests that the differential CPP performance between both mutant mice at that dose is not due to a differential locomotor performance deficits induced by cocaine. Overall, these results suggested that there was likely to be underlying differences in neuronal function caused by their respective genetic manipulation. This result may reflect the differences between the powerful effects of overexpressing a deletion mutation of BAF53b (*Baf53bΔHD*) compared with the loss of one *Baf53b* allele. It is plausible that a deletion mutant of BAF53b ultimately affects the nBAF complex in a persistent manner such that even higher cocaine doses are unable to induce strong CPP. The hydrophobic domain is probably forming important protein–protein interactions, which are then disrupted in the transgenic *Baf53bΔHD* mice. However, this possibility remains open to further exploration.

To further elucidate the underlying mechanisms that might account for the differences observed between the *Baf53bΔHD* and *Baf53b*^*+/−*^ heterozygous knockout, we developed a theta stimulation protocol to induce long-term synaptic changes at glutamatergic synapses in NAc slices. Theta-driven wave patterns are a natural occurrence in the rodent and occur during learning in the hippocampus^45^ and NAc^46^. We found that both mutant lines showed normal LTP induction. However, the level of potentiation in slices from *Baf53bΔHD* mice was significantly reduced, whereas *Baf53b*^*+/−*^ heterozygous knockout mice showed a slight attenuation in the level of LTP with respect to the WT group. In addition, these results also support the interpretation that the deletion mutation of BAF53b (*Baf53bΔHD*) leads to a significant LTP impairment compared with the heterozygous knockout and this profile was consistent with the cocaine dose–response differences observed between the two genetically modified mouse lines at 10 mg kg^−1^ in the CPP experiments.

BDNF is a neuromodulator released from terminals following TBS and supports LTP^38^,^39^,^40^,^41^,^42^. In addition, BDNF/TrkB signalling has been implicated in drug-context memory and drug-associated behaviours in the NAc^46^,^47^,^48^,^49^,^50^,^51^,^52^,^53^. Therefore, even though BDNF/TrkB signalling has an established role in theta-LTP (in the hippocampus^41^,^54^,^55^ and drug-associated memories (in the NAc)), there were open questions as to whether BDNF/TrkB receptor activation is required for synaptic plasticity in the NAc and capable of ameliorating LTP and CPP deficits induced by a BAF53b mutation. The current study determined that TrkB receptor activation is required for theta-LTP in the NAc using ANA12, a TrkB receptor antagonist^43^. We observed that infusions of ANA12 blocked theta-LTP in a reversible manner. Bath-applied BDNF generated a rapid increase in synaptic transmission in slices from both WT and *Baf53bΔHD* mice. This BDNF-induced increase in synaptic transmission was blocked by ANA12, indicating that BDNF effects were being modulated through the TrkB receptor.

On examining the effects of bath-applied human BDNF on both WT and mutant slices, we found that BDNF restored the ability of *Baf53bΔHD* slices to maintain a similar level of potentiation as found in WT slices. Given that WT slices did not display potentiation above that of *Baf53bΔHD* slices after bath BDNF application, we interpret this to mean that BDNF influence on the stabilization of LTP in the mutants probably involves events that are initiated after induction, which is consistent with our observation that short-term potentiation was unaffected in these mice. As described in an earlier report, BAF53b appears to be involved in activity-induced actin signalling in hippocampus CA1 pyramidal cells^23^. Additional morphological and cell signalling studies should reveal whether the same holds true for BAF53b in NAc neurons. Taken together, our results suggest that BDNF/TrkB receptor activation is integral to theta-LTP in the NAc and it is possible that there is a role for BAF53b in BDNF-related signalling cascades. The interaction between BAF53b and BDNF/TrkB signalling needs to be further explored; however, this effort is currently hampered by the unavailability of a chromatin immunoprecipitation-grade BAF53b antibody.

The successful rescue of LTP in slices from mutant mice using an acute application of human recombinant BDNF led us to explore a possible rescue of the CPP impairments in these mutant mice. To deliver a sustained amount of BDNF that could last over the course of a CPP experiment, we used an adeno-associated virus overexpressing human BDNF. Delivery of this virus into the striatum has previously been shown to ameliorate the cognitive deficits in a transgenic model of dementia, while rescuing motor deficits^56^. We pursued a rescue of the *Baf53bΔHD* mice, because they displayed the most severe LTP and CPP deficits. Our results showed that overexpressing BDNF in the NAc was sufficient to overcome CPP deficits in *Baf53bΔHD* mice. This result suggests that the deficits initially observed in *Baf53bΔHD* mice were not due to developmental confounds, because the *Baf53bΔHD* mice with overexpressing BDNF were capable of establishing a CPP.

Consistent with the behavioural data, viral overexpression of BDNF in *Baf53bΔHD* mice was able to restore theta-LTP to potentiated levels found in slices from WT+mCherry mice. Notably, the level of potentiation was dramatically increased following TBS in slices from WT+BDNF mice when compared with WT+mCherry mice. Furthermore, WT+BDNF group slice displayed potentiation significantly above that of the *Baf53bΔHD*+BDNF group. This result was not predicted as we observed that after bath application of BNDF, potentiation was comparable in slices taken from WT and mutant mice. This discrepancy in the level of LTP is possibly due to the differential effects of acute (bath applied) versus chronic (viral overexpressed) human BDNF exposure. That is, chronic elevations in BDNF, along with activity-dependent release of the neurotrophin, may have set in motion long-term plasticity changes in the NAc including an increase in dendritic spine density and in the number of synapses^57^,^58^,^59^ that would, on one hand, amplify long-term plasticity under normal conditions, whereas, on the other hand, normalize aberrant spines as to produce control levels of LTP. In contrast, the introduction of a brief infusion of BDNF in a NAc slice was enough to cause an immediate positive change in baseline synaptic function for both groups that then led to a saturation of theta-LTP in WT slices, while overcoming the LTP deficit in mutant slices. These results highlight the importance of evaluating the effect of BDNF/TrkB receptor activation on LTP using a variety of experimental approaches.

In summary, recent studies have implicated the BAF complex and its subunits in severe intellectual disability disorders and, more recently, the neuron-specific and dedicated subunit of the nBAF complex, BAF53b, has been shown to be critical for long-lasting forms of synaptic plasticity and long-term memory^23^. Considering the dynamic interplay between histone modification mechanisms, which have a key role in regulating gene expression induced by cocaine and other drugs of abuse, and nucleosome remodelling complexes, it is important to investigate the role of nucleosome remodelling complexes in cocaine-associated behaviours and memories. The present study extends the previous research by showing for the first time that neuron-specific nucleosome remodelling is critical for synaptic plasticity necessary for cocaine-associated memory formation and suggests studying neurotrophin receptors as a potential target to relieve BAF53b-associated deficits. Nucleosome remodelling complexes provide an additional layer of complexity to the epigenetic mechanisms that regulate gene expression induced by drugs of abuse and potentially provide novel possibilities for therapeutic avenues.

## Methods

### Mice

For all experiments, mice of either sex were between 8 and 15 weeks old at the time of behavioural testing. Mice had free access to food and water, and lights were maintained on a 12:12 h light per dark cycle, with all behavioural testing performed during the light portion of the cycle. Experimenters were blinded to genotype during behavioural and electrophysiological experiments. In addition, mice were genotyped after testing using DNA from tail samples. All experiments were conducted according to US National Institutes of Health Guidelines for Animal Care and Use, and were approved by the Institutional Animal Care and Use Committee of the University of California, Irvine.

### Immunoprecipitation and western blotting

WT *Baf53b* and *Baf53bΔHD* were cloned into pLVX-EF1a-IRES-mCherry (Clontech, Mountain View, CA) creating plasmids MW95 and MW97, respectively. HT22 cells (Salk Institute, La Jolla, CA) were transfected with either the empty vector, pLVX-EF1a-IRES-mCherry, MW95 or MW97 using Lipofectamine LTX with Plus Reagent (ThermoFisher, Waltham, MA). Cells were harvested 48 h after transfection and lysed in TPER (ThermoFisher). Immunoprecipitations against Brg1 were performed using 1 μg of antibody ab70558 (Abcam,, Cambridge, MA). Western blottings were probed for BAF53b (75–311) at a 1:1,000 dilution (Neuromab, Davis, CA) and for Brg1 at a 1:1,000 dilution using the same antibody as above. Western blot images have been cropped for presentation. Full-size images are presented in Supplementary Fig. 3.

### Immunofluorescence

WT mice were killed through cervical dislocation and their brains removed and frozen by immersion into isopentane cooled to ∼−50 °C on dry ice. Coronal sections were then cut at a thickness of 20 μm on a cryostat at the level corresponding to NAc (NAc; AP: +1.4 to +0.6 mm), thaw mounted on glass slides and stored at −20 °C until used for immunofluorescent staining. Immunofluorescence was carried out on the fresh–frozen, 20-μm thaw-mounted sections using the Tyramide Signal Amplification (TSA) Plus system (Perkin Elmer, NEL704A001KT). In brief, all slides were fixed using ice-cold 4% paraformaldehyde pH 7.4 (10 min), permeablized in 0.01% Triton X-100 in 0.1 M PBS (5 min) and incubated in 2% hydrogen peroxide diluted in 0.1 M PBS (vol/vol) (15 min). Subsequently, all slides were then blocked (1 h) using the TSA blocking buffer and incubated overnight (4 °C) in rabbit antibody to BDNF (1:500, Santa Cruz Biotechnology, sc-546), washed (3 × 5 min in 0.1 M PBS with 0.05% Tween-20, vol/vol) and incubated for 1 h at 21–23 °C with horseradish peroxidase-conjugated goat antibody to rabbit IgG (1:500, 711-036-152, Jackson ImmunoResearch). Slides were again washed and the TSA reaction was performed to detect BDNF as described in the kit (TSA-Alexa Fluor 488 1:50). Slides were coverslipped in ProLong Gold antifade reagent with DAPI (P-36931, Invitrogen).

### Quantitative real-time RT–PCR

Real-time RT–qPCR was performed to examine gene expression. Tissue was collected as described in our previous studies^6^,^9^. Tissue from the NAc was stored in RNALater (Invitrogen, AM7020) at −80 °C until processed. RNA was isolated using RNeasy minikit (Qiagen). Complementary DNA was made from 50 to 150 ng of total RNA using the Transcriptor First Strand cDNA Synthesis kit (Roche Applied Science, 04379012001). Primers were derived from the Roche Universal Probe Library. *Gapdh*: forward primers (5′–3′), ATGGTGAAGGTCGGTGTGA ; reverse primers (3′–5′), AATCTCCACTTTGCCACTGC ; Probe binding sequence (5′- TGGCCGTATTGG -3′). *Baf53b* wt: forward primers (5′–3′), TCCTGCCTTCTTCTTATGCAA ; reverse primers (3′–5′), CCTGTGGAGCGTCCATTT ; Probe binding sequence 100 (5′- GCTCACAG -3′). *Baf53bΔHD*: forward primers (5′–3′), GGTTTTCCTCATTAAAGGCATT ; reverse primers (3′–5′), TTCCAACCTATGGAAGTGATG ; Probe binding sequence 32 (5′- CTGCTCCC -3′). *Gapdh* probe is conjugated to Lightcycler Yellow555 and all other probes were conjugated to a reporter fluorophore. The non-overlapping dyes and quencher on the reference gene allow for multiplexing in the Roche LightCycle 480 II machine (Roche Applied Science). All values were normalized to *Gapdh* expression levels. Analysis and statistics were performed using the Roche proprietary algorithms and REST 2009 software based on the Pfaffl method.

### Cocaine sensitization

This conditioning examines the locomotor activating effects of cocaine in animals due to acute or chronic cocaine administrations. In brief, before any behavioural procedure, mice were handled for 1–2 min for 3 days and were habituated to the activity apparatus (Plexiglas open field with sawdust bedding; base 16 cm × 32 cm) for 30 min per day for 2 consecutive days. Mutant mice and their respective WT littermates were randomized into three different treatment groups (Control, Acute and Chronic) and locomotor activity was monitored for 30 min after an intraperitoneal (i.p.) injection of cocaine-HCl (10 mg kg^−1^; Sigma) or vehicle (0.9% saline). The Control group received saline injections on days 1–5. The Acute group received saline injections on days 1–4 and a single cocaine injection on day 5. The Chronic group received cocaine injections on days 1–5. Locomotor activity (total distance travelled) was recorded each day with a video camera mounted above the activity apparatus and tracked automatically from MPEG videos using EthoVision 3.1 software (Noldus Technology, Leesburg, VA; see ref. 6).

### Conditioned place preference

Place conditioning was performed as described previously in our studies^6^,^9^. Briefly, all mice were handled for 1 min each day for 3 consecutive days before the experiment (days 1–3). Baseline preferences were assessed by placing the animals in the centre compartment of the three compartment place preference apparatus with the guillotine doors open, allowing free access to three distinct compartments for 15 min (day 4). Time spent in each compartment was recorded. Conditioning was conducted over the subsequent 4 days with the guillotine doors closed, thus confining animals to a specific compartment for 30 min (days 5–8). An unbiased paradigm was used such that half of the animals were injected with cocaine-HCl (5 or 10 mg kg^−1^, i.p.; Sigma) before placement in the checkered compartment and half were injected with cocaine before placement in the white compartment (CS+). The next day, mice were injected with 0.9% saline (1.0 ml kg^−1^, i.p.) before placement in the alternate compartment (CS−). Injections were alternated for subsequent conditioning sessions. Forty-eight hours after the last conditioning session, preference (15 min, Posttest 1; day 10) was assessed in all animals as described above in a drug-free state. CPP score was calculated as the time (s) spent in cocaine-paired (CS+) minus saline-paired (CS−) compartments. Time spent in each chamber of the CPP apparatus was tracked automatically from MPEG videos using EthoVision 3.1 software (Noldus Technology; see ref. 6).

### Slice preparation and recording

Parasagittal slices containing the NAc core were prepared from BAF53b^+/*−*^, CamKIIα-BAF53bHD^Low^ and WT mice (∼2 months of age). Following isoflurane anaesthesia, mice were decapitated and the brain was quickly removed and submerged in ice-cold, oxygenated dissection medium containing (in mM): 124 NaCl, 3 KCl, 1.25 KH_2_PO_4_, 5 MgSO_4_, 2.5 CaCl_2_, 26 NaHCO_3_ and 10 glucose. The cerebellum and lateral aspects of both hemispheres were removed. Parasagittal slices (320 μm) were cut from the blocked brain using a FHC vibrating tissue slicer (Model:OTS-5000) before being transferred to an interface recording chamber containing preheated artificial cerebrospinal fluid (aCSF) of the following composition (in mM): 124 NaCl, 3 KCl, 1.25 KH_2_PO_4_, 1.5 MgSO_4_, 2.5 CaCl_2_, 26 NaHCO_3_, 10 glucose and 10 μM picrotoxin to reduce feedforward inhibition (refs 56, 57, 58). Slices were continuously perfused with this solution at a rate of 1.0–1.5 ml min^−1^, while the surface of the slices were exposed to warm, humidified 95% O_2_/5% CO_2_ at 31±1 °C. Recordings began following at least 1.5 h of incubation.

Stimulation of glutamatergic afferent fibres within the NAc was achieved by placing a bipolar stainless steel stimulation electrode (25 μm diameter, FHC) just below the anterior commissure. Activation of fEPSPs were recorded using a glass pipette (2–3 MΩ) positioned caudal or caudal–ventral to the stimulation electrode (Fig. 4a). Thus, correct placement of electrodes within the NAc was confirmed by visual inspection of the slice and comparison with mouse brain atlas (Paxinos and Watson; 0.84–1.08 lateral to midline). Two parasagittal slices/hemisphere containing a large portion of the NAc core were obtained for each animal. Pulses were administered at 0.05 Hz using a current that elicited a 30–40% maximal response. Measurements of fEPSP slope (measured at 10–90% fall of the slope) were recorded during a minimum 20 min stable baseline period at which time LTP was induced by delivering three to five trains (intertrain interval of 1 min), each train containing five ‘theta' bursts, with each burst consisting of four pulses at 100 Hz and the bursts themselves separated by 200 ms (that is, TBS). The stimulation intensity was not increased during the delivery of TBS. Data were collected and digitized by NAC 2.0 Neurodata Acquisition System (Theta Burst Corp., Irvine, CA) and stored on a disk.

Data in the text are presented as means±s.d. Data in figures on LTP were normalized to the last 10 min of baseline. The LTP experiment and conventional measures of baseline synaptic transmission including paired-pulse facilitation and input/output curves were analysed using a two-way repeated measure analysis of variance.

### Reagents

The AMPA receptor antagonist, DNQX (Tocris) and human recombinant form of BDNF (Chemicon) were prepared fresh in aCSF, whereas the TrkB receptor antagonist, ANA12 (Tocris), stock was dissolved in dimethyl sulfoxide and then diluted to working concentration in aCSF (dimethyl sulfoxide <0.01%). BDNF virus (AAV2-CAG2-mCherry-2A-mBDNF-WPRE; AAV-253926) and control virus (AAV2-CAG2-mCherry-WPRE) were generous donations form Dr Mathew Blurton-Jones.

### Data analysis

For all experiments, no differences were observed with respect to sex; thus, data sets were collapsed. Data sets were then analysed by two-way repeated-measures analysis of variance. Bonferroni *post-hoc* tests were performed when appropriate. Specific comparisons were made using Student's *t*-tests with *α*-levels held at 0.05.

### Data availability

The data that support the findings of this study are available from the corresponding author on request.

## Additional information

**How to cite this article:** White, A. O. *et al*. BDNF rescues BAF53b-dependent synaptic plasticity and cocaine-associated memory in the nucleus accumbens. *Nat. Commun.* 7:11725 doi: 10.1038/ncomms11725 (2016).

## Supplementary Material

Supplementary Information Supplementary Figures 1 - 3

## References

[b1] Hyman S. E. Addiction: a disease of learning and memory. Am. J. Psychiatry 162, 1414–1422 (2005). 16055762 10.1176/appi.ajp.162.8.1414

[b2] Kalivas P. W., Szumlinski K. K. & Worley P. Homer2 gene deletion in mice produces a phenotype similar to chronic cocaine treated rats. Neurotox. Res. 6, 385–387 (2004). 15545022 10.1007/BF03033313

[b3] Shen H. Y. . Additive effects of histone deacetylase inhibitors and amphetamine on histone H4 acetylation, cAMP responsive element binding protein phosphorylation and DeltaFosB expression in the striatum and locomotor sensitization in mice. Neuroscience 157, 644–655 (2008). 18848971 10.1016/j.neuroscience.2008.09.019

[b4] Valjent E., Pagès C., Hervé D., Girault J. A. & Caboche J. Addictive and nonaddictive drugs induce distinct and specific patterns of ERK activation in mouse brain. Eur. J. Neurosci. 19, 1826–1836 (2004). 15078556 10.1111/j.1460-9568.2004.03278.x

[b5] Kumar A. . Chromatin remodeling is a key mechanism underlying cocaine-induced plasticity in striatum. Neuron 48, 303–314 (2005). 16242410 10.1016/j.neuron.2005.09.023

[b6] Malvaez M., Mhillaj E., Matheos D. P., Palmery M. & Wood M. A. CBP in the nucleus accumbens regulates cocaine-induced histone acetylation and is critical for cocaine- associated behaviors. J. Neurosci. 31, 16941–16948 (2011). 22114264 10.1523/JNEUROSCI.2747-11.2011 PMC3235434

[b7] Maze I. . Essentialrole of the histone methyltransferase G9a in cocaine-induced plasticity. Science 327, 213–216 (2010). 20056891 10.1126/science.1179438 PMC2820240

[b8] Renthal W. . Histone deacetylase 5 epigenetically controls behavioral adaptations to chronic emotional stimuli. Neuron 56, 517–529 (2007). 17988634 10.1016/j.neuron.2007.09.032

[b9] Rogge G. A., Singh H., Dang R. & Wood M. A. HDAC3 is a negative regulator of cocaine-context-associated memory formation. J. Neurosci. 33, 6623–6632 (2013). 23575859 10.1523/JNEUROSCI.4472-12.2013 PMC3670682

[b10] Schroeder F. A. . Drug-induced activation of dopamine D(1) receptor signaling and inhibition of class I/II histone deacetylase induce chromatin remodeling in reward circuitry and modulate cocaine-related behaviors. Neuropsychopharmacology 33, 2981–2992 (2008). 18288092 10.1038/npp.2008.15 PMC2746694

[b11] Sun J. . The effects of sodium butyrate, an inhibitor of histone deacetylase, on the cocaine- and sucrose maintained self-administration in rats. Neurosci. Lett. 441, 72–76 (2008). 18599214 10.1016/j.neulet.2008.05.010

[b12] White A. O. & Wood M. A. Does stress remove the HDAC brakes for the formation and persistence of long-term memory? Neurobiol. Learn. Mem. 112, 61–67 (2014). 24149059 10.1016/j.nlm.2013.10.007 PMC3992200

[b13] Malvaez M., Barrett R. M., Wood M. A. & Sanchis-Segura C. Epigenetic mechanisms underlying extinction of memory and drug-seeking behavior. Mamm. Genome 20, 612–623 (2009). 19789849 10.1007/s00335-009-9224-3 PMC3157916

[b14] Rogge G. A. & Wood M. A. The role of histone acetylation in cocaine-induced neural plasticity andbehavior. Neuropsychopharmacology 38, 94–110 (2013). 22910457 10.1038/npp.2012.154 PMC3521972

[b15] Nestler E. J. Epigenetic mechanisms of drug addiction. Neuropharmacology 76, Pt B 259–268 (2014). 23643695 10.1016/j.neuropharm.2013.04.004 PMC3766384

[b16] Jonkman S. & Kenny P. J. Molecular, cellular, and structural mechanisms of cocaine addiction: a key role for microRNAs. Neuropsychopharmacology 38, 198–211 (2013). 22968819 10.1038/npp.2012.120 PMC3521966

[b17] Romieu P. . Histone deacetylase inhibitors decrease cocaine but not sucrose self-administration in rats. J. Neurosci. 28, 9342–9348 (2008). 18799668 10.1523/JNEUROSCI.0379-08.2008 PMC6671106

[b18] Kennedy P. J. . Class I HDAC inhibition blocks cocaine-induced plasticity by targeted changes in histone methylation. Nat. Neurosci. 16, 434–440 (2013). 23475113 10.1038/nn.3354 PMC3609040

[b19] Hargreaves D. C. & Crabtree G. R. Genomics and Mechanisms pp 1–25Nature Publishing Group (2011).

[b20] Ho L. & Crabtree G. R. Chromatin remodeling during development. Nature 463, 474–484 (2010). 20110991 10.1038/nature08911 PMC3060774

[b21] Lessard J. . An essential switch in subunit composition of a chromatin remodeling complex during neural development. Neuron 55, 201–215 (2007). 17640523 10.1016/j.neuron.2007.06.019 PMC2674110

[b22] Wu J. I. . Regulation of dendritic development by neuron-specific chromatin remodeling complexes. Neuron 56, 94–108 (2007). 17920018 10.1016/j.neuron.2007.08.021

[b23] Vogel-Ciernia A. . The neuron-specific chromatin regulatory subunit BAF53b is necessary for synaptic plasticity and memory. Nat. Neurosci. 16, 552–561. 23525042 10.1038/nn.3359 PMC3777648

[b24] Zhao K. . Rapid and phosphoinositol-dependent binding of the SWI/SNF-like BAF complex to chromatin after T lymphocyte receptor signaling. Cell 95, 625–636 (1998). 9845365 10.1016/s0092-8674(00)81633-5

[b25] Olave I., Wang W., Xue Y., Kuo A. & Crabtree G. R. Identification of a polymorphic, neuron-specific chromatin remodeling complex. Genes Dev. 16, 2509–2517 (2002). 12368262 10.1101/gad.992102 PMC187451

[b26] Park J., Wood M. A. & Cole M. D. BAF53 forms distinct nuclear complexes and functions as a critical c-Myc-interacting nuclear cofactor for oncogenic transformation. Mol. Cell Biol. 22, 1307–1316 (2002). 11839798 10.1128/mcb.22.5.1307-1316.2002 PMC134713

[b27] Mayford M. . Control of memory formation through regulated expression of a CaMKII transgene. Science 274, 1678–1683 (1996). 8939850 10.1126/science.274.5293.1678

[b28] Kojima N. . Rescuing impairment of long-term potentiation in fyn-deficient mice by introducing Fyn transgene. Proc. Natl Acad. Sci. USA 94, 4761–4765 (1997). 9114065 10.1073/pnas.94.9.4761 PMC20798

[b29] Renthal W. . Genome-wide analysis of chromatin regulation by cocaine reveals a role for sirtuins. Neuron 62, 335–348 (2009). 19447090 10.1016/j.neuron.2009.03.026 PMC2779727

[b30] Kombian S. B. & Malenka R. C. Simultaneous LTP of non-NMDA—and LTD 55 of NMDA—receptor-mediated responses in the nucleus accumbens. Nature 368, 242–246 (1994). 7908412 10.1038/368242a0

[b31] Thomas M. J., Malenka R. C. & Bonci A. Modulation of long-term depression by dopamine in the mesolimbic system. J. Neurosci. 20, 5581–5586 (2000). 10908594 10.1523/JNEUROSCI.20-15-05581.2000 PMC6772537

[b32] d'Alcantara P., Ledent C., Swillens S. & Schiffmann S. N. Inactivation of adenosine A2A receptor impairs long-term potentiation in the accumbens nucleus without altering basal synaptic transmission. Neuroscience 107, 455–464 (2001). 11719000 10.1016/s0306-4522(01)00372-4

[b33] Schramm N. L., Egli R. E. & Winder D. G. LTP in the mouse nucleus accumbens is developmentally regulated. Synapse 45, 213–219 (2002). 12125042 10.1002/syn.10104

[b34] Mishra D., Zhang X. & Chergui K. Ethanol disrupts the mechanisms of induction of long-term potentiation in the mouse nucleus accumbens. Alcohol Clin. Exp. Res. 36, 2117–2125 (2012). 22551245 10.1111/j.1530-0277.2012.01824.x

[b35] Post R. M. & Kalivas P. Bipolar disorder and substance misuse: pathological and therapeutic implications of their comorbidity and cross-sensitisation. Br. J. Psychiatry 202, 172–176 (2013). 23457180 10.1192/bjp.bp.112.116855 PMC4340700

[b36] Russo S. J., Mazei-Robison M. S., Ables J. L. & Nestler E. J. Neurotrophic factors and structural plasticity in addiction. Neuropharmacology 56, (Suppl 1) 73–82 (2009). 18647613 10.1016/j.neuropharm.2008.06.059 PMC2635335

[b37] Balkowiec A. & Katz D. M. Cellular mechanisms regulating activity-dependent release of native brain-derived neurotrophic factor from hippocampal neurons. J. Neurosci. 22, 10399–10407 (2002). 12451139 10.1523/JNEUROSCI.22-23-10399.2002 PMC6758764

[b38] Aicardi G. . Induction of long-term potentiation and depression is reflected by corresponding changes in secretion of endogenous brain-derived neurotrophic factor. Proc. Natl Acad. Sci. USA 101, 15788–15792 (2004). 15505222 10.1073/pnas.0406960101 PMC524856

[b39] Figurov A., Pozzo-Miller L. D., Olafsson P., Wang T. & Lu B. Regulation of synaptic responses to high-frequency stimulation and LTP by neurotrophins in the hippocampus. Nature 381, 706–709 (1996). 8649517 10.1038/381706a0

[b40] Kramár E. A. . A novel mechanism for the facilitation of theta-induced long-term potentiation by brain-derived neurotrophic factor. J. Neurosci. 24, 5151–5161 (2004). 15175384 10.1523/JNEUROSCI.0800-04.2004 PMC6729196

[b41] Patterson S. L. . Recombinant BDNF rescues deficits in basal synaptic transmission and hippocampal LTP in BDNF knockout mice. Neuron 16, 1137–1145 (1996). 8663990 10.1016/s0896-6273(00)80140-3

[b42] Tyler W. J., Alonso M., Bramham C. R. & Pozzo-Miller L. D. From acquisition to consolidation: on the role of brain-derived neurotrophic factor signaling in hippocampal-dependent learning. Learn. Mem. 9, 224–237 (2002). 12359832 10.1101/lm.51202 PMC2806479

[b43] Cazorla M. . Identification of a low molecular weight TrkB antagonist with anxiolytic and antidepressant activity in mice. J. Clin. Invest. 121, 1846–1857 (2011). 21505263 10.1172/JCI43992 PMC3083767

[b44] Jia Y., Gall C. M. & Lynch G. Presynaptic BDNF promotes postsynaptic long-term potentiation in the dorsal striatum. J. Neurosci. 30, 14440–14445 (2010). 20980601 10.1523/JNEUROSCI.3310-10.2010 PMC2972744

[b45] Otto T., Eichenbaum H., Wiener S. I. & Wible C. G. Learning-related patterns of CA1 spike trains parallel stimulation parameters optimal for inducing hippocampal long-term potentiation. Hippocampus 1, 181–192 (1991). 1669292 10.1002/hipo.450010206

[b46] Donnelly N. A. . Oscillatory activity in the medial prefrontal cortex and nucleus accumbens correlates with impulsivity and reward outcome. PLoS ONE 9, e111300 (2014). 25333512 10.1371/journal.pone.0111300 PMC4205097

[b47] Bahi A., Boyer F., Chandrasekar V. & Dreyer J. L. Role of accumbens BDNF and TrkB in cocaine-induced psychomotor sensitization, conditioned-place preference, and reinstatement in rats. Psychopharmacology 199, 169–182 (2008). 18551281 10.1007/s00213-008-1164-1

[b48] Crooks K. R., Kleven D. T., Rodriguiz R. M., Wetsel W. C. & McNamara J. O. TrkB signaling is required for behavioral sensitization and conditioned place preference induced by a single injection of cocaine. Neuropharmacology 58, 1067–1077 (2010). 20176040 10.1016/j.neuropharm.2010.01.014 PMC3676179

[b49] Graham D. L. . Dynamic BDNF activity in nucleus accumbens with cocaine use increases self-administration and relapse. Nat. Neurosci. 10, 1029–1037 (2007). 17618281 10.1038/nn1929

[b50] Graham D. L. . Tropomyosin-related kinase B in the mesolimbic dopamine system: region-specific effects on cocaine reward. Biol. Psychiatry 65, 696–701 (2009). 18990365 10.1016/j.biopsych.2008.09.032 PMC2738869

[b51] Hall F. S., Drgonova J., Goeb M. & Uhl G. R. Reduced behavioral effects of cocaine in heterozygous brain-derived neurotrophic factor (BDNF) knockout mice. Neuropsychopharmacology 28, 1485–1490 (2003). 12784114 10.1038/sj.npp.1300192

[b52] Horger F. S., Drgonova J., Goeb M. & Uhl G. R. Reduced behavioral effects of cocaine in heterozygous brain-derived neurotrophic factor (BDNF) knockout mice. Neuropsychopharmacology 28, 1485–1490 (1999). 10.1038/sj.npp.1300192 12784114

[b53] Lobo M. K. . Cell type-specific loss of BDNF signaling mimics optogenetic control of cocaine reward. Science 330, 385–390 (2010). 20947769 10.1126/science.1188472 PMC3011229

[b54] Korte M. . Virus-mediated gene transfer into hippocampal CA1 region restores long-term potentiation in brain-derived neurotrophic factor mutant mice. Proc. Natl Acad. Sci. USA 93, 12547–12552 (1996). 8901619 10.1073/pnas.93.22.12547 PMC38029

[b55] Korte M. . Hippocampal long-term potentiation is impaired in mice lacking brain-derived neurotrophic factor. Proc. Natl Acad. Sci. USA 92, 8856–8860 (1995). 7568031 10.1073/pnas.92.19.8856 PMC41066

[b56] Goldberg N. R. . Neural stem cells rescue cognitive and motor dysfunction in a transgenic model of dementia with lewy bodies through a BDNF-dependent mechanism. Stem Cell Rep. 5, 791–804 (2015). 10.1016/j.stemcr.2015.09.008 PMC4649255 26489892

[b57] Tyler W. J. & Pozzo-Miller L. D. BDNF enhances quantal neurotransmitter release and increases the number of docked vesicles at the active zones of hippocampal excitatory synapses. J. Neurosci. 21, 4249–4258 (2001). 11404410 10.1523/JNEUROSCI.21-12-04249.2001 PMC2806848

[b58] Tyler W. J. & Pozzo-Miller L. Miniature synaptic transmission and BDNF modulate dendritic spine growth and form in rat CA1 neurones. J. Physiol. 553, Pt 2 497–509 (2003). 14500767 10.1113/jphysiol.2003.052639 PMC2343578

[b59] Kellner Y. . The BDNF effects on dendritic spines of mature hippocampal neurons depend on neuronal activity. Front. Synaptic Neurosci. 6, 5 (2014). 24688467 10.3389/fnsyn.2014.00005 PMC3960490

