# Supplementary material for: BDNF rescues BAF53b-dependent synaptic plasticity and cocaine-associated memory in the nucleus accumbens
Source: Nat Commun. 2016 May 26;7:11725. doi: 10.1038/ncomms11725 (PMC4894971; doi:10.1038/ncomms11725)
Supplement: Supplementary Information — Supplementary Figures 1 - 3 [file ncomms11725-s1.pdf]

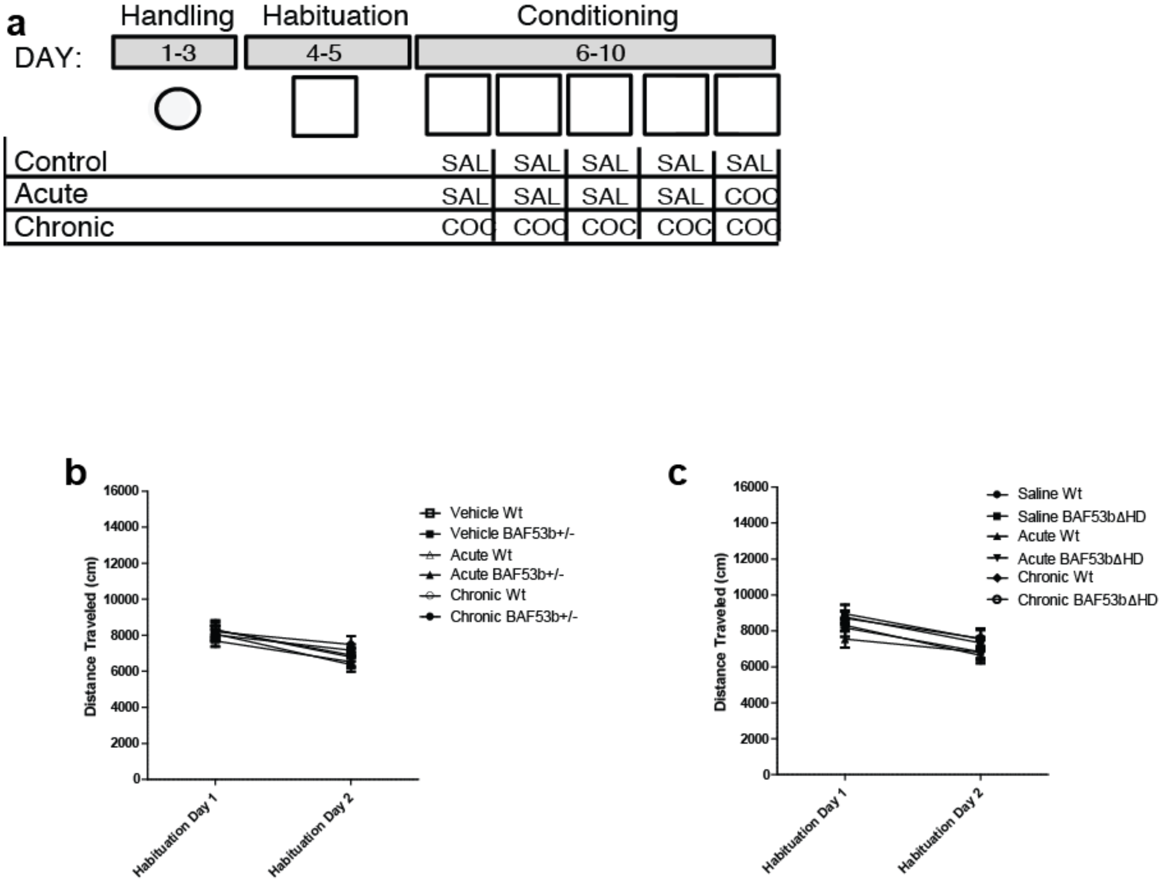

Supplementary Figure 1. BAF53b$^{+/-}$ heterozygous knockout and BAF53bΔHD mice displayed normal baseline locomotor activity during habituation. **(a)** Schematic representation of cocaine sensitization procedure. **(b)** BAF53b$^{+/-}$ heterozygous knockout and wildtype mice, regardless of future treatment, displayed similar locomotor activity throughout habituation (significant main effect of habituation day, $F_{1,24}=69.79$, *p<0.0001;* no main effect of genotype, $F_{2,24}=0.41$, *p=0.67*; no interaction, $F_{2,24}=0.91$, *p=0.41)*. **(c)** BAF53bΔHD and wild-type mice displayed similar locomotor activity throughout habituation (significant main effect of habituation day, $F_{1,20}=37.08$, *p<0.0001;* no main effect of genotype, $F_{2,20}=2.02$, *p=0.16*; no interaction, $F_{2,20}=1.38$, *p=0.27)*.

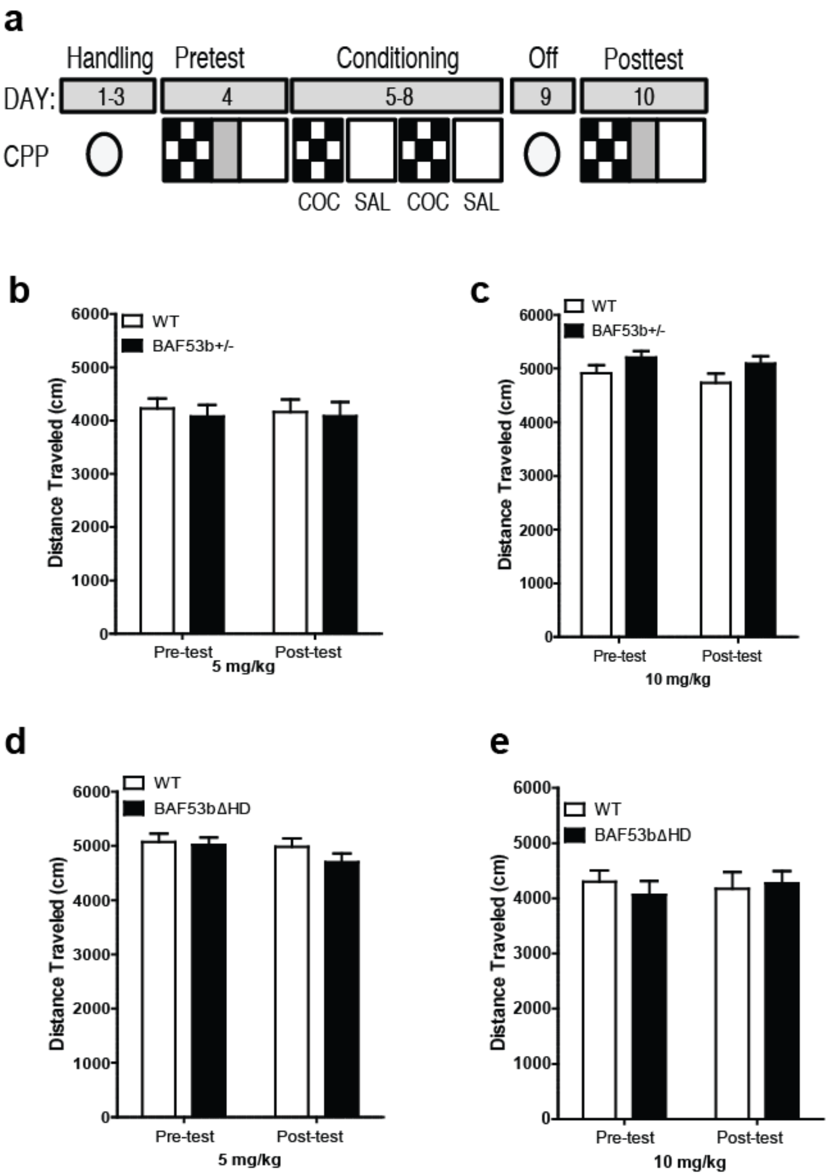

Supplementary Figure 2. Mutant mice have normal locomotion on test days during cocaine-CPP. **(a)** Schematic representation of cocaine-CPP procedure. **(b)** Cocaine-CPP expression indicated by mean CPP score (CS+ minus CS−) ± S.E.M. At 5mg/kg cocaine dose, BAF53b$^{+/-}$ heterozygous knockout mice (n=10) exhibited similar locomotion compared to wild-type littermates (n=8). A two-way repeated measures ANOVA revealed no main effect of genotype ($F_{1,16}$=0.14, *p=0.72)*. No effect of conditioning was observed ($F_{1,16}$=*0.09, p=0.77)* and there was no interaction ($F_{1,16}$=0.16, *p=0.69)*. **(c)** At 10mg/kg cocaine dose, BAF53b$^{+/-}$ heterozygous knockout mice (n=9) exhibit similar CPP score to wild-type littermates (n=8). Using a two-way repeated measures ANOVA, we found no main effect of conditioning ($F_{1,17}$=2.22, *p=0.15)* nor genotype ($F_{1,17}$=3.07, *p=0.09)* and no interaction ($F_{1,17}$=0.09, *p=0.77*). **(d)** At 5mg/kg cocaine dose, BAF53bΔHD mice (n=9) exhibited significantly attenuated CPP score compared to wild-

type littermates (n=10). Using a two-way repeated measures ANOVA, we found significant main effects on conditioning ($F_{1,17}$=5.20, *p=0.03*) but not genotype ($F_{1,17}$= 0.70, *p=0.42*), and no interaction ($F_{1,17}$=1.68, *p=0.21*). **(e)** At a 10mg/kg cocaine dose, BAF53bΔHD mice (n=10) exhibited significantly attenuated CPP score compared to wild-type littermates (n=7).). A two-way repeated measures ANOVA revealed significant main effects of conditioning ($F_{1,15}$=0.08, *p=0.78)*, genotype ($F_{1,15}$=0.05, *p=0.83)* and an interaction ($F_{1,15}$=1.30, *p=0.27)*.

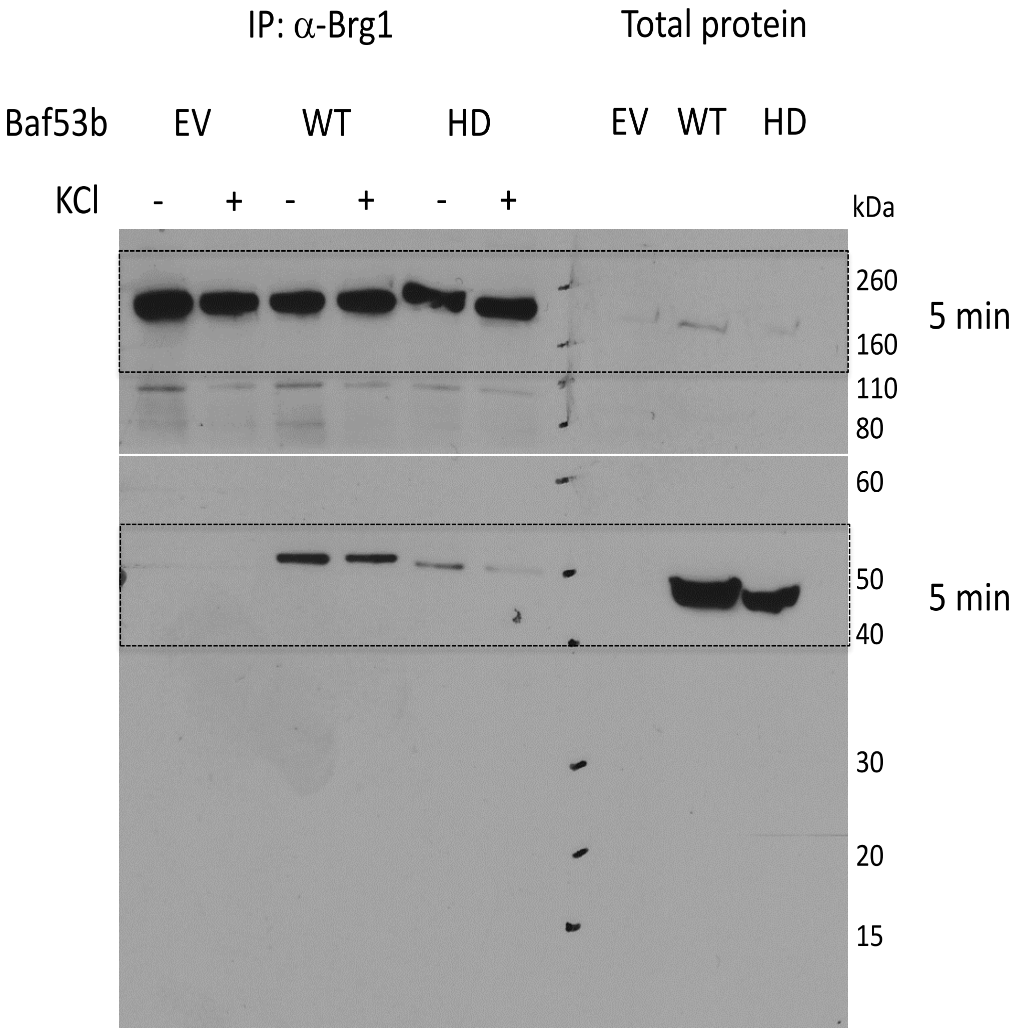

Supplementary Figure 3. Immunoprecipitation and western blot showing that Brg1 (top dotted box) co-immunoprecipitates with BAF53b (Bottom dotted box) as well as BAF53bΔHD. Image attained through 5 minute exposure.
